# Whale Shark (*Rhincodon typus*) Seasonal Presence, Residence Time and Habitat Use at Darwin Island, Galapagos Marine Reserve

**DOI:** 10.1371/journal.pone.0115946

**Published:** 2014-12-31

**Authors:** David Acuña-Marrero, Jesús Jiménez, Franz Smith, Paul F. Doherty, Alex Hearn, Jonathan R. Green, Jules Paredes-Jarrín, Pelayo Salinas-de-León

**Affiliations:** 1 Charles Darwin Research Station, Puerto Ayora, Galapagos Islands, Ecuador; 2 Department of Fish, Wildlife, and Conservation Biology, Colorado State University, Fort Collins, Colorado, United States of America; 3 Turtle Island Restoration Network, Olema, California, United States of America; 4 Galapagos National Park Directorate, Puerto Ayora, Galapagos Islands, Ecuador; University of California Davis, United States of America

## Abstract

The life history of the whale shark (*Rhincodon typus*), including its reproductive ecology, still remains largely unknown. Here, we present results from the first whale shark population study around Darwin Island, Galapagos Marine Reserve. Following a diversified approach we characterized seasonal occurrence, population structure and size, and described habitat use of whale sharks based on fine scale movements around the island. Whale shark presence at Darwin Island was negatively correlated with Sea Surface Temperature (SST), with highest abundance corresponding to a cool season between July and December over six years of monitoring. From 2011 to 2013 we photo-identified 82 whale sharks ranging from 4 to 13.1 m Total Length (TL). Size distribution was bimodal, with a great majority (91.5%) of adult female individuals averaging 11.35 m±0.12 m (TL±SE), all but one showing signs of a potential pregnancy. Population dynamics models for apparently pregnant sharks estimated the presence of 3.76±0.90 (mean ± SE) sharks in the study area per day with an individual residence time of 2.09±0.51 (mean ± SE) days. Movement patterns analysis of four apparently pregnant individuals tracked with acoustic tags at Darwin Island revealed an intense use of Darwin's Arch, where no feeding or specific behavior has been recorded, together with periodic excursions around the island's vicinity. Sharks showed a preference for intermediate depths (20–30 m) with occasional dives mostly to mid-water, remaining the majority of their time at water temperatures between 24–25°C. All of our results point to Darwin Island as an important stopover in a migration, possibly with reproductive purposes, rather than an aggregation site. Current studies carried out in this area to investigate regional scale movement patterns may provide essential information about possible pupping grounds for this enigmatic species.

## Introduction

The whale shark, *Rhincodon typus* Smith 1828, has a widespread global distribution, occurring throughout the tropical and sub-tropical seas [1]. *Rhincodon typus* is classified since 2002 as Vulnerable by the International Union for the Conservation of Nature (IUCN), mainly due to declining populations as revealed by decreasing landings in whale shark fisheries in the Pacific and Indian oceans [2]. Despite its global ecological and socio-economical significance, much of its life history, including its reproductive ecology and migratory routes, remains largely unknown [3].

Whale sharks are known to segregate by sex and size [3]–[8] and in recent decades several seasonal aggregations have been identified, providing opportunities for a number of studies focused on this species [8]–[12]. Rowat and Brooks [3] described an aggregation as a site with more than 10 individuals in less than 1 km^2^. Most of these aggregations comprised immature males (6–8 m Total Length (TL)) and they appear to exist mostly for feeding purposes. Therefore certain aspects of whale shark's biology and ecology remain poorly understood, especially those related to reproduction [3], [5], [6], [13]. Adult female (≥9 m TL) [1] and neonate (<1 m TL) published records are scarce, and apparently pregnant females have been only reported consistently in the Gulf of California [7], [14]–[16], Galapagos Islands [17] and Philippines [3]. Additionally, Hueter et al. [18] described a 7.5 m TL female tagged off Isla Holbox (Mexican Caribbean) in 2007, with a pelvic region that was “noticeable enlarged”, although the size at maturity for female whale sharks is estimated at 9 m TL [1]. Hueter et al. [18] did not confirm this individual's (named Rio Lady) maturity or pregnancy, but hypothesized that the satellite track recorded from the Gulf of Mexico to the center of the Atlantic Ocean (7,772 km) could have been to give birth. Sequeira et al. [19] suggested that large-scale whale shark migrations have different patterns depending on sex and size, and that females may display natal philopatry, given that the longest satellite tracks recorded to date all belong to large females. If this was the case, mature females would carry out long migrations to the same breeding grounds where they were born, so studies focusing on pregnant whale sharks could provide essential information to corroborate this hypothesis.

Habitat use information for this highly mobile and migratory species has been studied by both satellite and acoustic telemetry [20], [21] and also through photo-identification (photo-ID) studies [16]. Continuous tracking using acoustic telemetry provides fine-scale information about habitat use that cannot be obtained in such detail by the use of satellite tags, as the latter relies on data transmission when the tag is at the surface (e.g., SPOT 5, SPLASH); or provide average daily positions with large error margins, when the tag has been released once its programmed time of deployment is completed (e.g., Pop-up Archival Transmitting (PAT) tags). To date, only Gunn et al. [20] have published a study based on tagging whale sharks with acoustic tags and carried out a continuous tracking of this species, at Ningaloo Reef, Western Australia. Gunn et al. [20] showed that *R. typus* diving behavior seems to be adapted to environmental and bathymetric features, with a diel diving pattern comprising deeper dives during the day and shallow dives at night [3], [4], [20]. This diving pattern would match the vertical migrations of zooplankton [4]. However, opposite diel diving patterns have also been documented for some individuals in coastal waters, indicating possible diving pattern variations as a function of local conditions [3], [22].

Spot patterns in *R. typus* are a distinctive individual feature, and photos from the lateral area behind the fifth gill are used to identify individual sharks [23]–[25]. Wildbook for Whale Sharks, the global whale shark library (https://www.whaleshark.org), provides two independent algorithms to find potential matches and re-sightings within its extensive database [11], [24], [25]. This non-invasive photo-ID technique allows for population size and residence time estimations in mark-recapture studies and to establish connectivity between different locations [3], [8], [10], [16], [19].

In some locations whale sharks are known to occur on a predictable and seasonal basis, including the Galapagos Islands. The first individual in the Archipelago was recorded north of Fernandina Island by early explorer William Beebe during the Arcturus expedition in 1925 [26], [27]. Other early sighting records were all from the central part of the Archipelago [26]–[30], and it was not until the mid 1980s when recreational SCUBA divers began to visit the northern islands of Wolf and Darwin, that whale sharks were reported from the northern part of Galapagos (Green pers. observ.).

In the present study we provide the first quantitative characterization of whale sharks' presence around Darwin Island, Galapagos Marine Reserve. The main objectives of this study were: 1) to document whale shark occurrence, seasonality, population structure and residence time around Darwin Island; and 2) to investigate local movement patterns to infer habitat usage around this isolated oceanic island.

## Materials and Methods

### Ethics statement

This research was approved by the Galapagos National Park Directorate as part of the 2013 Operational Plan of the Charles Darwin Foundation, with the methods described here reviewed and approved by a Galapagos National Park Directorate's committee that assess animal care in research activities.

### Study site

**Figure 1:**
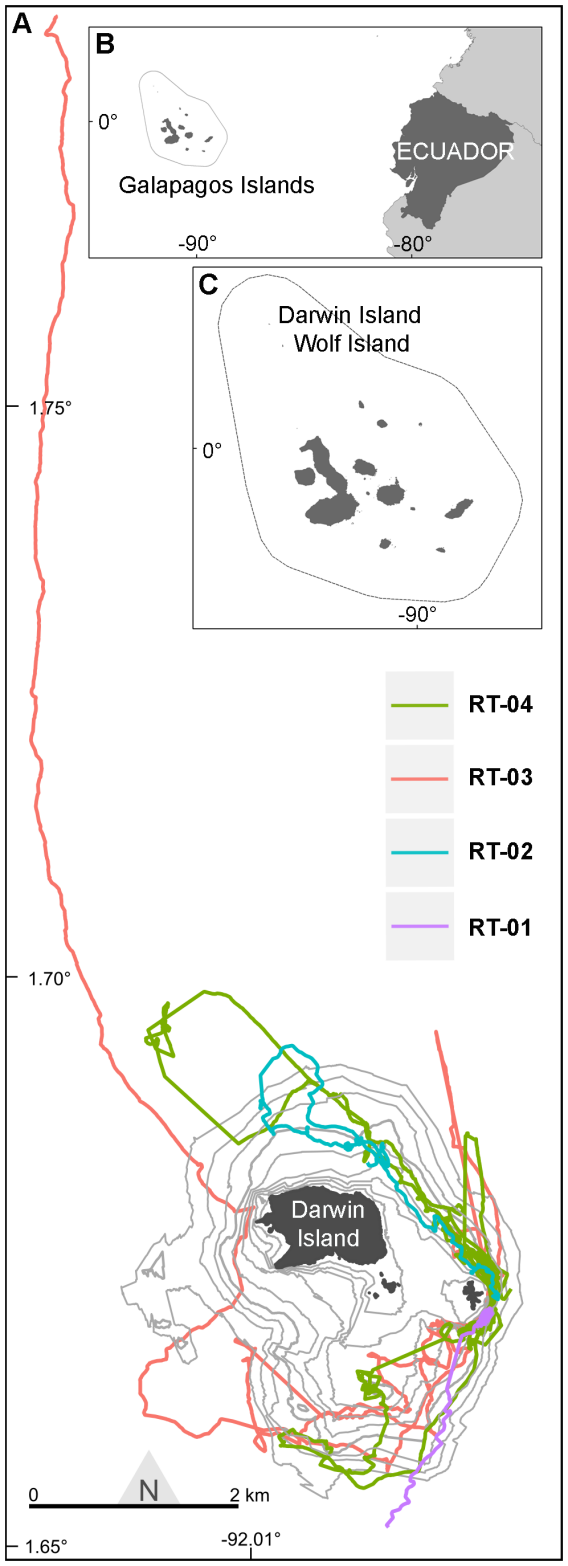
Location of the whale shark study area. A) Tracks of the four *R. typus* around Darwin Island with its bathymetry (10 m isobaths); B) Galapagos Archipelago's setting in the Eastern Tropical Pacific (Ecuador national territory in dark grey); C) Galapagos Islands with the Galapagos Marine Reserve boundaries that extends 40 nm from the islands (discontinued line).

The Galapagos Islands comprise a group of 13 major islands and over 100 islets and rocks situated around the equator at approximately 1,000 km off mainland Ecuador, in the Eastern Tropical Pacific region (Fig. 1) [31]. The Archipelago possesses a unique and highly variable oceanographic setting, as it is located in the confluence of three major oceanic currents, proximate to the Equatorial Front (EF) and exposed to upwelling conditions [32].

Darwin is the northernmost of the Galapagos Islands and, together with its neighbor Wolf Island, makes up the Far North bio-geographical region, largely based on reef fish and macroinvertebrate assemblages (Fig. 1) [33]. This area is influenced by the Panama Current from the northeast, and is the warmest region of the Archipelago [34]. Darwin is a steep and rocky volcanic island, with a submerged rocky platform at a depth of about 10 m and extending approximately one mile to the southeast between the island and a small (0.2 ha) rocky emergent arch, Darwin's Arch (1°40.41′N 91°59.35′W; Fig. 1). The island-platform-arch unit is surrounded by a steep slope that descends to depths greater than 100 m [35]. The site is exposed to the predominant southeastern current and home to an extraordinarily high density of multiple species of sharks and other reef and pelagic fish that change on a seasonal basis [36], [37].

### Data collection

#### Seasonality

We obtained Sea Surface Temperature (SST) data from weekly MODIS/Aqua 0.05-degree resolution data (https://modis.gsfc.nasa.gov/) over the observation period. We averaged SST data within 20 km of Darwin's Arch to characterize the general oceanographic conditions surrounding the island.

To determine *R.typus* seasonal presence at Darwin Island we analyzed a pelagic species monitoring database that comprises a total of 197 underwater visual surveys at the study site between November 2007 and November 2013, during both the warm and cool seasons. Surveys were conducted by a pair of divers at 15–25 m of depth. No whale shark photo-ID, sex determination nor size estimation was conducted during these surveys. Additionally, we reviewed the dates of all whale shark encounters from this area reported in the Wildbook for Whale Sharks database and got dive guides reports for over the past 20 years in order to complement the visual census information.

#### Population structure

We conducted a total of 180 dives focused on *R. typus* at the study site during several field trips per year for the duration of the study between July 2011 and November 2013 (cool season). Trips ranged between 5 and 15 days, with a team of 2–4 divers conducting three dives (45–50 min) per day (early morning, midday and afternoon). During each census we recorded information about shark's size, sex, signs of potential pregnancy (a clear distended belly, as described by Ramírez-Macías et al. [16]), presence of significant scars, behavior and associated fauna.

We determined the size of individual sharks using the laser photogrammetry technique as first described by Rohner et al. [38]. When photos could not be taken, we visually estimated TL.

#### Photo-identification

We identified individual sharks using left side photographs covering the area behind the fifth gill, given that the whale shark's left side has been standardized in most photo-ID studies [4], [8], [16]. Additionally, we also took right-side and prominent scar photographs to assist with the re-sighting analysis. We analyzed our photographs with the specifically dedicated Interactive Individual Identification System software (I3S, [25]), compiling them in a database to compare between individuals in search of matches/re-sightings. We also included encounters and photo-ID records from the study site submitted by the general public and researchers since 2002 to the Wildbook for Whale Sharks library (https://www.whaleshark.org) in our analysis.

#### Habitat use

We externally tagged four large (10–12 m TL), apparently pregnant whale sharks with continuous transmitters (V16TP-3x, Vemco, Ltd.). Transmitters were equipped with temperature and pressure (i.e., depth) sensors. We attached transmitters with a tether to a stainless steel dart (Wildlife Computers, Ltd.) and then we inserted the dart into the dorsal musculature that surrounds the first dorsal fin of the shark using pneumatic guns (Cressi Sub SL 100, Cressi Ltd.). Data were transmitted every 3000 ms and recorded by a VR100 unit (Vemco Ltd.) equipped with a directional hydrophone VH 110 (Vemco, Ltd.). We set gain of the VR100 (lowest possible), signal strength (highest possible), and direction to try to keep the skiff as close to the shark as possible, while attempting to maintain its same swimming speed. For safety reasons, our tracking activities were restricted to a maximum of five nautical miles away from Darwin Island.

### Data analysis

#### Seasonality and population structure

We combined data from visual observations with SST values during the dates closest to the observation date (i.e. usually within the week). In periods where no SST data were available (e.g. during periods of cloud cover), we took the closest SST value from the MODIS/Aqua satellite as the best estimate of the SST during the observation period.

We used oneway analysis of variance (ANOVA) to test for monthly differences in SST between 2007–2013. We compared sightings per hour between seasons (warm/cool) and size distributions obtained by laser photogrammetry and visual estimation using the Wilcoxon Rank Sum test. We conducted all statistical analysis using the software R (The R Foundation for Statistical Computing).

#### Intra-seasonal abundance and residence time

We analyzed the sighting records of 47 sharks identified during a sampling trip in September 2012 and a trip in October 2012. We excluded from our analysis the two immature sharks recorded in this period to meet the model assumptions (below). Both sampling trips had similar duration (11–10 days, respectively), a constant sampling effort (a team of four divers performing three 40 min dives per day: early morning, mid-day and evening), and were carried out during the cool season, with the trip dates selected randomly within this season.

To estimate the superpopulation of whale sharks in an open population model that does not assume demographic closure, we used the POPAN model [39], [40] as implemented in Program MARK [41], [42]. We interpreted superpopulation as the number of whale sharks that passed through the sampled area during each of our sampling trips. This model allows the estimation of the superpopulation (N), detection probabilities (p_t_) for each day (t), apparent survival (φ_t_) for each interval between days and the probability of entry into the sampled population for each day (β_t_). We analyzed each trip separately. We considered the parameters p, t, φ, and β to be either constant across time or to vary by time leading to a set of 8 models. We relied upon Akaike’s information criteria with a small sample size correction (AICc) for model selection [43].

The model is based on several assumptions, as described in Schwarz and Anarson [42]. To meet these assumptions: 1) we attempted to photograph every individual we sighted; 2) since sampling intervals (i.e., a single day) were short, we assumed that daily survival was equal to 1, and that any disappearance of individuals was due to permanent emigration and that this emigration rate was the same for all individuals; 3) we assumed that all pregnant females in the region follow a similar movement pattern that takes them through Darwin Island; 4) since we relied upon the self-marking patterns of the whale sharks, mark loss was not an issue, and we only used photographs in which we were confident of identification; 5) we kept our sampling intervals short, as they were during a single day; 6) we rarely saw an individual whale shark for more than two days in a row and thus emigration was probably permanent; 7) we conducted a goodness-of-fit test using the median *ĉ* procedure. There is no goodness-of-fit (GOF) test for the full POPAN model, so we relied upon the simpler Cormack-Jolly-Seber (CJS) model for our GOF test and adjusted metrics and estimates as needed.

Since our sampling trips were 10-11 days and our sampling intervals were <1 day, we assumed that mortality over this time was zero and that all disappearance represented emigration. We thus could interpret apparent survival (φ) as the probability of remaining in the sampled area (i.e., 1- probability of emigration) and estimate residence time. Specifically, we used the Method 2 formulas (which are available in the help file in Program MARK) that are associated with Kendall and Bjorkland [44] model to calculate residence time. This formula is:
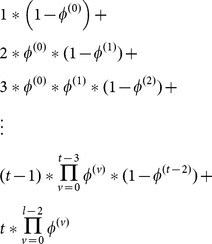



Where 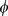 is the probability that an individual in the study area at time t is still in the study area at time t+1 (i.e., apparent survival) and v is the number of time periods between arrival and t.

We also estimated the total intra-seasonal (Jul-Dec) abundance of pregnant whale sharks that moved past Darwin Island. For this analysis we needed to assume: a constant rate of *R. typus* survival and probability of entry at study site (derived from the 21 days of sampling) for the entire 184 days of this period; and a constant rate of observation (through photo-ID) during surveys. We also set observation equal to zero for days with no surveys.

#### Habitat use

We filtered all depth and water temperature measurements provided by the transmitters to remove false values following this process: 1) we removed all negative depth and temperature values and those out of the range of the transmitter specification; 2) we took out vertical movements if rate of depth change exceeded 2 m/s, as the maximum vertical velocity and total movement rate recorded by this species during continuous tracking studies were not higher than 1.4 and 1.8 m/s, respectively [20], [45]; 3) we removed depth values significantly higher than bottom depth values for the same position.

To obtain bottom depth profile for the tracks we used a Darwin Island Bathymetry [35] resampled to 5 m resolution.

We joined all tracks obtained to analyze whale shark spatial use at Darwin Island, as their reduced number and high variability in duration prevented us from analyzing them separately. We estimated the Utilization Distribution (UD) [46], which is the probability density to relocate the animal at any place of the study site according to its coordinates [47]. To estimate the UD we used the kernel method, as proposed by Worton [48]. To achieve this, we used the KernelUD function provided by the package adehabitatHR in R (The R Foundation for Statistical Computing). Before running this algorithm we merged the whole datasets to have a general overview of most frequently used areas. Our aim was to conduct a spatial analysis in a broad scale in order to determine if whale sharks spend significant amount of time in other areas of Darwin Island apart from Darwin's Arch. For this reason we did not find necessary to subsample in longer intervals to avoid or reduce possible boat movements not related with the shark, as described in Klimley et al. [49]. We used the complete GPS dataset (GPS allocation rate of 3 sec) to estimate home range. The KernelUD smoothing parameter was determined manually by selecting different bandwidths and cell sizes until the most appropriate was found that could be used to explore high densities space used, as suggested by Silverman [50]. We obtained the best satisfactory results with a bandwidth of 100 and a cell size of 5 m, allowing us to have a good insight of hotspots covering mainly eastern side of Darwin's Arch. In addition, we deduced home range from UD, covering the minimum area on which the probability to relocate the animals is equal to 0.95 and 0.50, respectively.

## Results

### Seasonality

**Figure 2:**
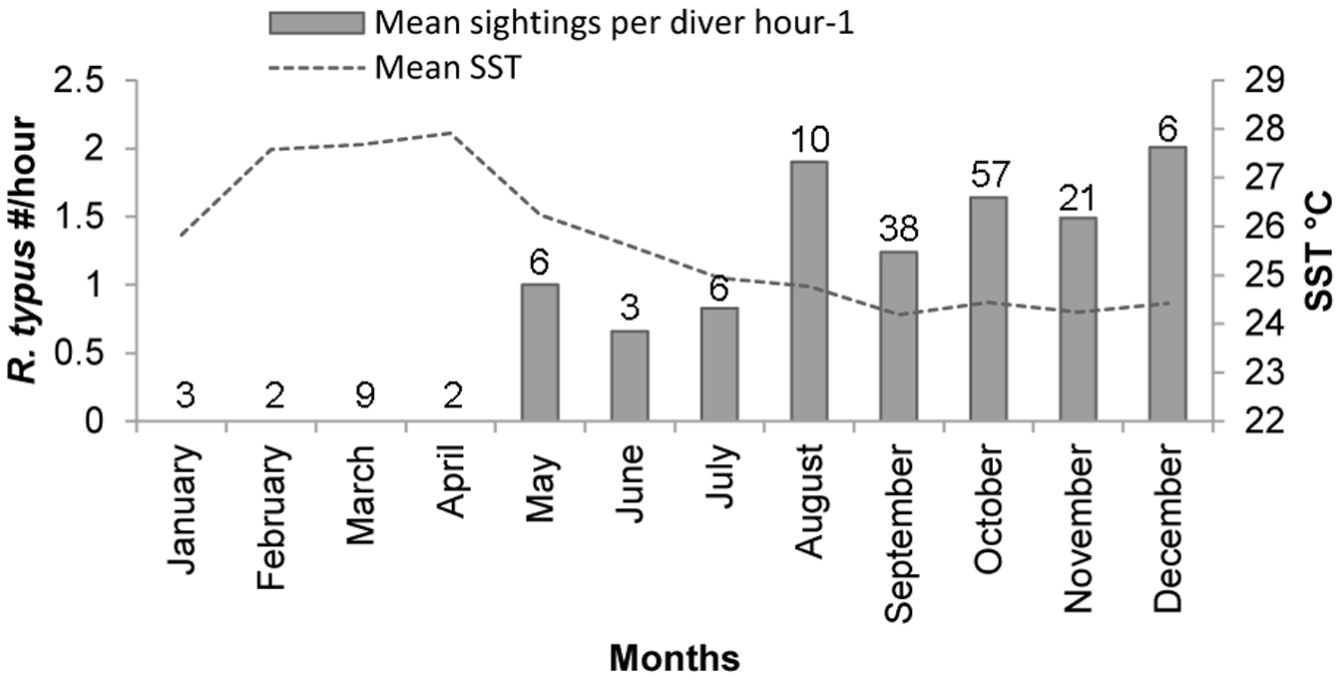
Monthly distribution of *R. typus* at Darwin Island. Plot showing whale shark sightings per diver hour^−1^ (left axis, grey bars) with mean Sea Surface Temperature (SST; right axis, grey dotted line) at Darwin Island, Galapagos Marine Reserve, between 2007–2013. Values over columns indicate the sampling effort (i.e., number of surveys) for each month.

Mean SST around Darwin Island was significantly different between months between 2007 and 2013 (Oneway ANOVA, p<.0001). Only between July and December does mean SST remains below 25°C (Fig. 2). Based on these results and in order to conduct our analysis and provide comparisons with other studies we considered two different seasons at Darwin Island: a warm season from January to June, and a cool season from July to December.

On the 197 censuses carried out at Darwin's Arch between 2007 and 2013 (23.2% in warm season, 86.8% in cool season) we recorded a total of 211 whale sharks sightings. Four of them corresponded to the months of May and June, while the rest of the records (207) were made between July and December, with no whale sharks recorded between January and April (Fig. 2). The number of *R. typus* sighted per hour was significantly different (Wilcoxon  = 2484, p<0.001) between warm (0.30±0.14, mean ± SE) and cool (1.51±0.17, mean ± SE) seasons. In the Wildbook for Whale Sharks database we found only one sighting during the core of the warm season (Feb-Apr) corresponding to an immature (4.2 m TL) recorded in February 2010 (Wildbook for Whale Sharks), while dive guides reported very rare sightings during these months, all corresponding to juvenile sharks (Morán pers. comm.).

**Figure 3:**
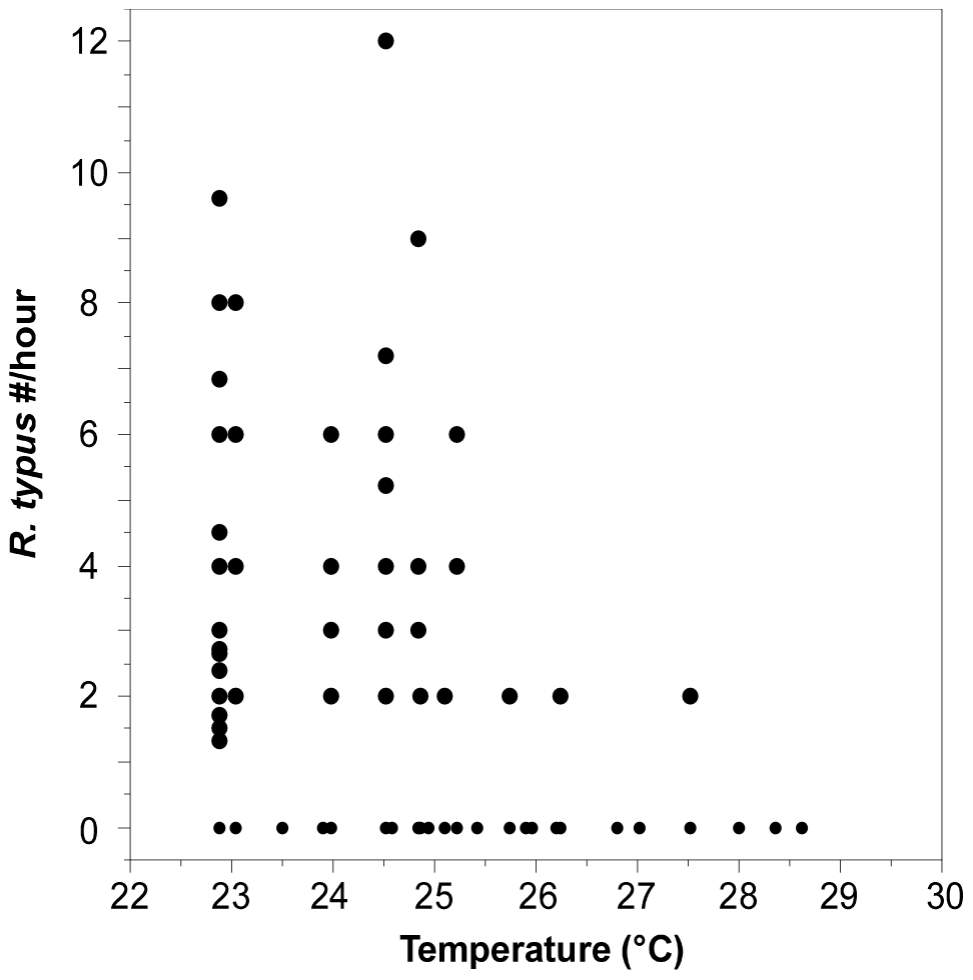
Relationship between SST and sighting frequency of *R. typus*. Least square regression's result between SST and whale shark sightings per diver hour^−1^ in the vicinity of Darwin's Arch from November 2007 to December 2013.

Patterns of sighting frequency of *R. typus* with respect to temperature showed a general decreasing trend with increasing temperature (least squares regression slope  = -0.8; Fig. 3). Although the data were concentrated in the cooler months, the data suggest the greatest sighting frequency occurs in the range of 23 to 25°C, where average number of *R. typus* sightings per hour was 0.26 (±0.03SE). This represents an approximate 1.4-fold greater sighting frequency during cool season from the average recorded by us at Darwin Island (i.e., mean  = 0.18±0.02, SE).

### Population structure

**Figure 4:**
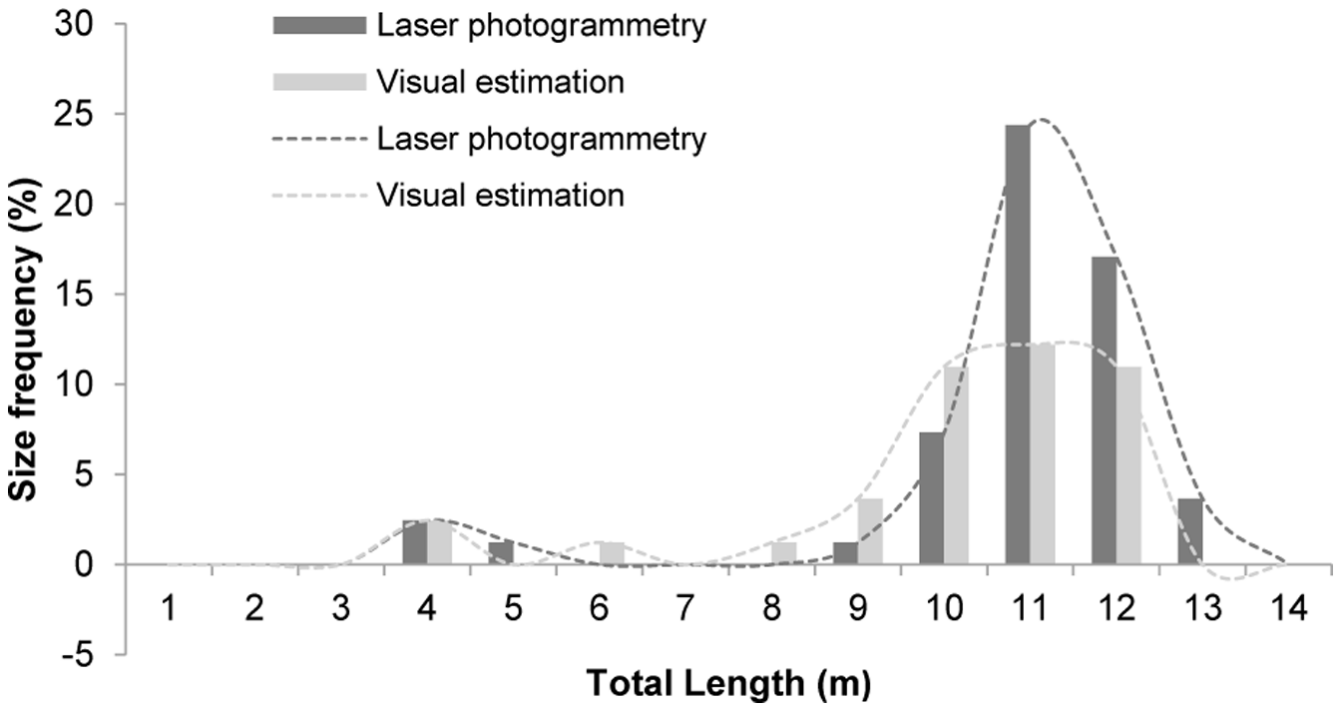
Size-frequency distribution. Size measurements by laser photogrammetry (N = 47; dark grey bars and dotted line) and visual estimation (N = 35; light grey bars and dotted line) of the 82 whale sharks photo-identified at Darwin Island, Galapagos Marine Reserve, between 2011 and 2013.

**Figure 5:**
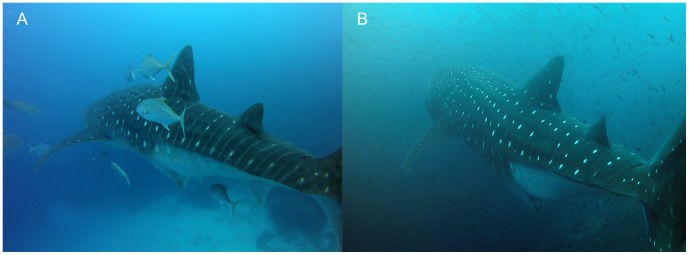
Visual evidence of *R. typus* possible pregnancy. Image of two female whale sharks recorded at Darwin Island that displayed different signs of pregnancy (A) a 9.3 m TL female sighted on November 18^th^ 2013 with its belly not distended; (B) a 12 m TL female sighted on October 20^th^ 2012 with a clear distended belly, which is a sign of pregnancy (Ramírez-Macías et al. 2012b); (photo credit: David Acuña-Marrero).

Of the 82 individual whale sharks that we identified and sexed at Darwin's Arch between July 2011 and November 2013, all but one were females. We measured 47 individuals using laser photogrammetry, while we visually estimated the size of the remaining 35 individuals. Although the mean TL obtained by laser photogrammetry and visual estimation measurements were significantly different (W = 1224.5, p-value <0.001), both methodologies provided similar distribution curves (Fig. 4). Assuming laser photogrammetry as the most accurate methodology, we detected an observer tendency to underestimate TL measurements, with an error lower than a meter, in our visual estimations in sizes over 9 m (Fig. 4). The size distribution of sharks was bimodal (Fig. 4). Shark sizes ranged from 4 to 13.1 m TL, represented by 8.5% of immature sharks ranging from 4 to 8 m TL (5.33±0.56, mean ± SE), including the only male recorded (14% of total immature sharks; 6.5 m TL), and 91.5% of adult females from 9 to 13.1 m TL (11.35±0.12, mean ± SE). Of the total number of sharks identified, 75 female sharks were above size of maturity (≥9 m TL) [1], and only one of them did not show a swollen abdomen (9.3 m TL measured by laser photogrammetry; Fig. 5A), while the rest of them had clear distended bellies (Fig. 5B), which could be a sign of pregnancy [16].

### Intra-seasonal abundance and residence time

From a total of 201 encounters with whale sharks recorded by the authors at Darwin's Arch between July 2011 and November 2013, we identified 82 different *R. typus* individuals in 142 different encounters. Of these, only 12 (14.6%) sharks were re-sighted after being recorded for the first time, 6 (7.3%) were only re-sighted the day after its first record, and 6 (7.3%) during the next 2–7 days. Only one shark was re-sighted between years. Of our 82 identified individuals, 57 could be uploaded to the Wildbook for Whale Sharks global database, which already included another 65 sharks identified from the Galapagos Islands between 2002 and 2013. This search for intra and inter-annual re-sightings using this global database resulted in zero matches.

During the September and October 2012 field trips we observed 27 and 20 different apparently pregnant individuals, respectively. Of these 47 sharks, 34 (72.3%) were only recorded during one day, while of the other 13, 10 of them (21.3%) were re-sighted only the day after its first record, and three (6.4%) several days (3–7 days) after its first record. None of the sharks were re-sighted between these two trips.

Our GOF testing did not indicate any lack of fit as all estimates of c-hat were <1. The model that considered p, t, φ, and β as constants described the data better than more complicated models for both the first trip (ΔAICc>8) and the second trip (ΔAICc>21). Because all the weight of evidence was on this model, we relied on this model for our estimates (Table 1).

**Table 1:** Estimates of superpopulation, probability of detection, apparent survival, probability of entry and residence time for 47 whale sharks recorded at Darwin Island, Galapagos Marine Reserve, during two individual survey trips in September and October 2013.

	Estimate	SE	95% LCL	95% UCL
First Trip (September 3^rd^-13^th^)				
Superpopulation (N)	59.40	15.52	35.89	98.30
Probability of detection (p)	0.39	0.15	0.15	0.69
Apparent survival (φ)	0.57	0.11	0.35	0.77
Probability of entry (β)	0.09	0.01	0.07	0.10
Residence time (days)	2.35	1.53	1.14	3.56
Second Trip (October 12^th^–21^st^)				
Superpopulation (N)	44.48	14.91	23.46	84.33
Probability of detection (p)	0.48	0.24	0.12	0.86
Apparent survival (φ)	0.46	0.14	0.22	0.73
Probability of entry (β)	0.10	0.01	0.08	0.12
Residence time (days)	1.86	0.49	0.89	2.83

Over the entire cool season our model estimated an average of close to four whale sharks in the study area per day (3.76±0.90, SE) with a residence time of around two days (2.09±0.51, SE). The probability of observing a shark during the day (if present) was slightly less than 40% (0.39±0.12, SE), while the probability of a shark staying from one day to another was about 50% (0.53±0.09, SE). Considering the uncertainty around the estimates, the probability of observation, apparent survival, probability of entry, and residence time did not differ greatly between the two trips (Table 1). If we assume constancy in these rates for the entire cool season, we can estimate a net abundance of 695±166 (SE; 95%CI 442–1110) apparently pregnant whale sharks per season. We note that this estimate is based on a total of 21 days of surveys on either end of a 184 day period (Table 1) and this estimate should be viewed with caution.

### Habitat use

We tracked four apparently pregnant individuals tagged for periods between 3 hrs 15 min and 25 hrs 17 min (Table 2). All sharks were tagged at Darwin's Arch, therefore all tracks were initiated at this location.

**Table 2:** Summary of *R. typus* tracks at Darwin Island in 2013.

Shark ID	Total length (m)	Sex (apparently pregnant)	Start		Finish		Track duration (hh: mm)	Track length (m)	Mean depth, SE (m)	Min. depth (m)	Max. depth (m)	Surface time % (D/N)
			Date	Time	Date	Time						
RT-01	12	Female (yes)	07-Oct	15∶45	07-Oct	19∶06	3∶21	8,897.60	28.02±0.13	0.8	51.9	0.00/09.33
RT-02	12	Female (yes)	08-Oct	8∶52	08-Oct	12∶08	3∶15	8,996.51	26.31±0.20	1.1	84.1	0.77/0.00
RT-03	10	Female (yes)	14-Nov	10∶58	15-Nov	17∶39	15∶12	43,653.49	18.71±0.17	0.2	226.1	21.55/11.73
RT-04	11.8	Female (yes)	16-Nov	11∶19	17-Nov	23∶06	25∶17	49,775.92	23.89±0.07	0.3	67.8	3.11/2.16

**Figure 6:**
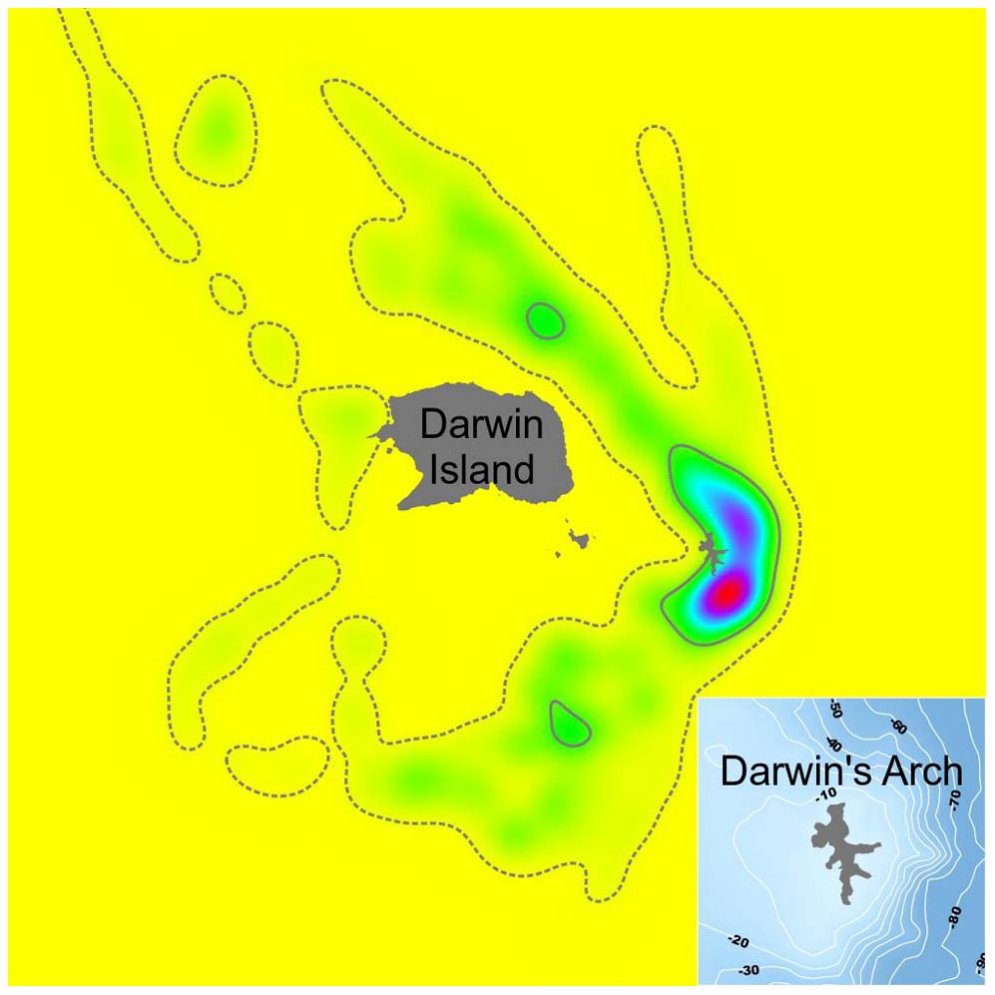
Kernel Utilization Distribution (UD) of whale sharks at Darwin Island. Kernel density is color coded, with warmer colors indicating highest probabilities and colder colors lower ones (A). Home range deduced from UD with 95% and 50% of probability indicated by dotted and continuous lines, respectively (A). (B) Detail of bathymetry at Darwin's Arch.

All shark movements were restricted to a distance of approximately 2.4 km around Darwin Island and its shallow platform (Fig. 6), with sharks only leaving this area upon apparently departing from the island, as RT-01 and RT-03 did (Fig. 1A). The 95% UD of the four whale sharks around the Island covered the north, east and south sides of the island, while 50% UD was restricted to an area mostly located around the east side of Darwin's Arch (Fig. 6).

**Figure 7:**
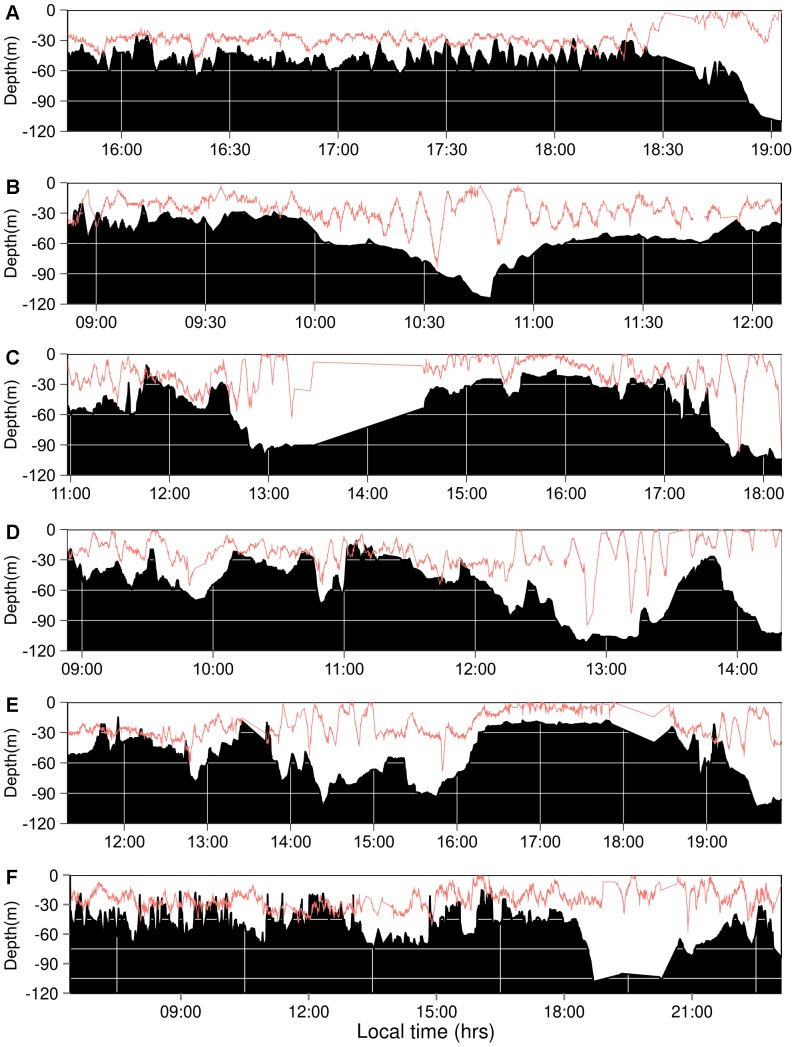
Whale sharks dive profiles at Darwin Island. Plots showing dive profile and bottom depth of RT-01 (A), RT-02 (B), RT-03 (C: Nov 14^th^, and D: Nov 15^th^, the latter only showing her dive profile during the time she remained in the vicinity of the island (<1.3 nm)) and RT-04 (E: Nov 16^th^, and F: Nov 17^th^), respectively. Signal from RT-03 was lost from 13:33 PM to 14:32 PM on Nov 14^th^ (C). Note that bottom depth contour was obtained from skiff position, so sharks dive's and bottom depth's profiles relationship may not coincide exactly with the reality. For this reason shark dive profiles overlap with bottom depth in few occasions, especially at shallow areas (<30 m).

**Figure 8:**
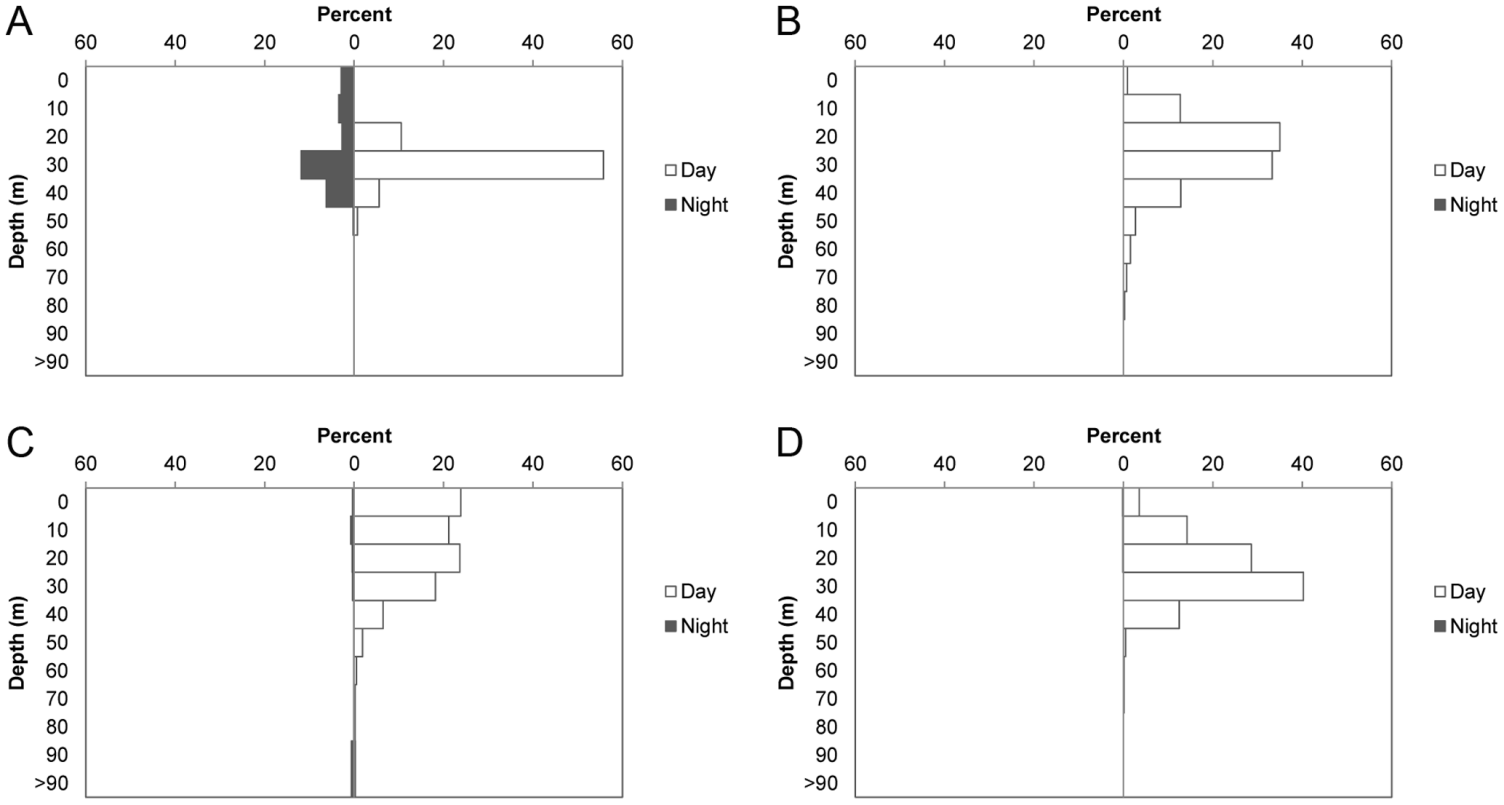
Diel depth preferences for whale sharks at Darwin Island. Percentages of time expended by each shark tracked (RT-01 (A), RT-02 (B), RT-03 (C) and RT-04 (D)) at different depth ranges during day and night hours.

**Figure 9:**
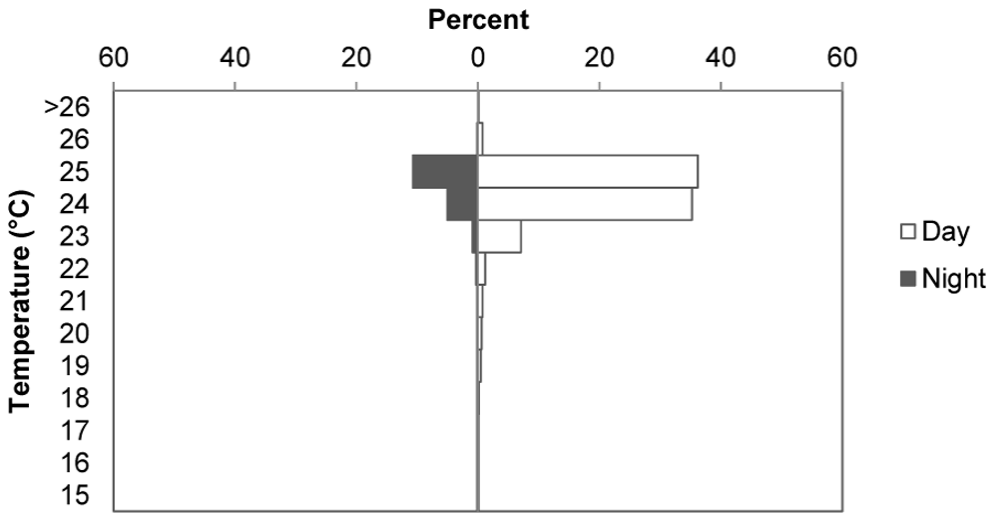
Diel temperature preferences for whale sharks at Darwin Island. Percent of time expended by the four sharks tracked at different water temperature during day and night hours.

The four sharks tracked showed similar mean depths that ranged 20–30 m, with the exception of RT-03, whose mean depth was slightly shallower (Table 2; Figs. 7 and 8). However, depth during the time she remained within the vicinity of the island (<2.4 km) was also 20–30 m (21.29±0.19, mean ± SE), and only changed to less than 5 m once she was on her way from the island (4.68±0.19, mean ± SE). Time spent at the surface was much reduced, or even non-existent, for the other three sharks and showed similar low values during day and night (Table 2). RT-03 was also the shark showing the deepest dive, reaching over 200 m, while the rest remained most of the time at depths of 10–50 m (Table 2). Bounce dives, a series of continued vertical oscillations >10 m [45], were restricted to a short series of dives (<5), mostly when bottom depth was over 60 m (Fig. 7). At shallower depths sharks' dive profiles remained roughly associated with the irregular bottom depth, performing occasional bottom bounce dives (Fig. 7) [45]. Whale sharks spent more than 95% of their time at temperatures of 23–25°C (Fig. 9).

## Discussion

The presence of *R. typus* at Darwin Island for the study period was strongly associated with oceanographic conditions, as most records were restricted to the cool season (Jul-Dec), when waters around the island reach their lowest SST values (23–25°C; Figs. 2 and 3). At this time of the year the cool Humboldt Current increases in intensity and the EF moves north and settles just north of Darwin Island [32]. This peak of whale shark abundance was followed by a clear decrease of sightings from the beginning of the warm season onwards (Fig. 2), when the warm Panama Current prevails and the EF moves south. Although our lower sampling effort during this season could have reduced our capacity to detect whale sharks, the general lack of whale shark sightings during those months has been also corroborated by records from the global Wildbook for Whale Sharks database and reports from local dive guides (Morán pers. comm.).

The sex ratio and size structure of *R. typus* at Darwin Island is clearly dominated by large females (≥9 m TL) showing clear distended bellies that could indicate a gravid status, with a minority group of immature females ranging 4–8 m TL. From a total of 482 encounters recorded during this study and those reported in the Wildbook for Whale Sharks database for Galapagos between 1999 and 2013, only two males, corresponding to two immature individuals (6.5 m TL in 2011 and 7.5 m TL in 2008; Wildbook for Whale Sharks, 2014), have been reported. Therefore the presence of male whale sharks at Darwin Island may be considered exceptional. Our data supports previous studies that indicate a pronounced segregation by sex for this species [3], [7]. To date, most information regarding *R. typus* population structure comes from coastal aggregations at different locations in the Caribbean Sea, Indian Ocean and the Gulf of California, and these aggregations are dominated by immature males [7], [9], [10], [12], [51], [52]. In contrast, Galapagos and the southern of the Gulf of California, both in the Tropical Eastern Pacific, are to date the only known locations where consistent sightings of large apparently pregnant individuals have been confirmed [6], [7], [14]–[17]. However, the presence of possibly pregnant individuals at both locations does not coincide in time nor oceanographic conditions. In the Gulf of California apparently pregnant female sightings occur during the spring months (Apr-Jun), which marks the transition between coolest winter SST to the highest SST of the summer [7], [16]. In Galapagos the presence of apparently pregnant females covers a much longer period, between May and December, and has its peak in fall, when the water reaches its lowest temperatures [32]. All *R. typus* observed in Galapagos Islands that are within the size of sexual maturity estimated for this species (≥9 m TL) [1] have shown potential signs of pregnancy, with the exception of one 9.3 m TL female recorded in November 2013. The difference in the shape and size of the abdomen of this individual compared with the apparently pregnant ones of similar TL was visually noticeable (Fig. 5). Whether this female was still immature, mature but not pregnant, in an initial phase of pregnancy not visually noticeable, or passed by Darwin after giving birth is not known, but in any case represents a single sighting at Darwin Island. To date, no study has confirmed the gravid condition of free-swimming females beyond visual inspection, since this represents a considerable logistical challenge. Future studies that focus on hormonal analysis of blood samples obtained from these apparently gravid individuals will provide very valuable information, although attempts of obtain blood samples from a free swimming individual in the wild have not been published thus far.

Our modeling results using program MARK revealed an intra-seasonal population dynamic characterized by a constant presence of whale sharks, with a high turnover rate in the number of individuals: Darwin Island is seasonally visited by a considerable number of apparently pregnant female whale sharks per day that remain in the island for a short period of time. This residence time at Darwin Island was lower in comparison with values reported at other locations that varied from 11–12 days at Utila Island, Honduras [8]; 35 days at Ningaloo Reef, Australia [11]; or up to 60 days in Bahía La Paz, Baja California, Mexico [16]. Identifying lack of fit with a GOF test is difficult with low sample size (i.e., only 10% of the season was sampled); thus our estimates of residence time should be viewed with caution. Also, our total intra-seasonal abundance result is subject to certain strong assumptions (as described in Results section) that need to be tested surveying through the entire season (184 days). In addition, data used for the estimation was only from 2012, therefore inter-annual variability still needs to be assessed. However, reports from local experienced dive guides point to a consistent intra-seasonal pattern of high whale shark occurrence during the second part of the year, with the only exception being the last strong ENSO (El Niño Southern Oscillation) event recorded in 1997–98 [34], during which period whale sharks were absent of Darwin Island, returning again in 2000 (Green pers. comm.).

This study provides the first evidence of local-scale movements of possible pregnant whale sharks. There is a recognizable pattern of spatial use at Darwin Island, characterized by an intense use of Darwin's Arch with apparently random excursions around other parts of the island, with no other hotspot of activity identified (Fig. 6). However, the reduced number of individuals tracked and the short duration of two of these tracks prevented us from considering our tracking study as a representative sample for the whole visiting population, although it may serve as a good indicator of their activity around the island. This intense use of the southeast of Darwin Island by apparently pregnant whale sharks is consistent with the results obtained by Hearn et al. [36] and Ketchum et al. [53] that reported a more intensive habitat use by scalloped hammerheads (*Sphyrna lewini*) and other pelagic species. Hearn et al. [36] and Ketchum et al. [53] provide several hypotheses to help explain this pattern, which include that this area: acts as landmarks with particular properties, such as magnetic field intensity, that could be used for orientation during seasonal migrations; constitutes a vantage location for nightly foraging excursions; and/or serves as cleaning stations, due to the abundance and diversity of other fish. Given that no feeding or cleaning behavior for whale sharks has ever been observed at Darwin Island, the first hypotheses may help explain whale sharks' heavy usage of this part of the island. However, while the ability to use the earth's magnetic field for navigational purposes has been confirmed in several shark species, the capacity of *R. typus* in this regard has yet to be demonstrated [3]. It could also be possible that, due to our limited tracking time at night, foraging night excursions from the study site could be undetected by us. However, their short residency time makes very unlikely that Darwin Island constitutes an important feeding ground for this species that justify their constant presence during the cool season.

The vertical movements of apparently pregnant whale sharks around the island were mostly limited to depths below 20 m with occasional short series of bounce dives to mid-water (Fig. 7). Very little time was spent at surface in this area, but this tendency changed once the shark left the island. This was the case of RT-03, who clearly changed her diving pattern and navigated most of the time at the surface when it followed a course away from Darwin, moving north. At Ningaloo Reef, Gunn et al. [20] found a more dispersed spatial use consisting of long-shore movements along the inner portion of the continental shelf, with the sharks carrying out long series of bounce dives to the bottom and spending a significant portion of their time at the surface, especially at night. Our tracking time at night was very limited (17% of our total tracking time; Table 2), but none of the sharks we tracked spent more time at the surface during nighttime. Ningaloo Reef is a well-known seasonal feeding ground for whale sharks and the horizontal and vertical movements of the sharks tracked there were associated with the search for food [20], [45]. These clear differences in spatial use and diving diel pattern between both locations, together with the lack of feeding observations at our study site, clearly suggest that the presence of apparently pregnant whale sharks at Darwin Island is not related to feeding. This is also supported by their short residence time at the island compared to other areas where sharks are known to aggregate to feed [8], [11], [16]. The possibility of an unknown site within the vicinity of the island where sharks might carry out feeding or other specific behavior seems unlikely, as our tracks indicate that sharks do not spend a significant amount of time in another specific site in Darwin Island aside from Darwin's Arch (Fig. 6). This lack of feeding or other specific behavior together with the apparently gravid condition of almost all adult female whale sharks observed indicate that their pass through Darwin Island in the case of adult individuals could be related to reproductive purposes. A similar pattern seems to be the case of the Gulf of California, where neither Eckert and Stewart [14] nor Ramírez-Macías et al. [16] recorded any activity or behavior by apparently pregnant females, while Ketchum et al. [7] only observed occasional ram-filtering feeding activity in some of their sightings but with a low abundance of potential prey in those areas.

In the case of the few immature females recorded, their low rate of sightings suggests an occasional pass through Darwin Island. Some authors have indicated that females may feed on more pelagic prey as this strategy will allow them to grow faster and reach a larger maturity size [3], [4], [7]. This could be why immature males are more common in coastal aggregations [3], leaving Darwin, an oceanic island located more than 1,000 km from the continental coast, as the only known site where individual immature sightings are female-biased.

## Conclusion

The lack of evidence of specific behavior observed at Darwin Island together with the short residence time and strong intra-seasonal abundance and high turnover rate indicate that this location is not an aggregation site but an important stopover in a migration. In the case of adult *R. typus* individuals observed, this migration probably has reproductive purposes, as all but one were apparently gravid. As many aspects of *R. typus* reproduction remain unknown, there is a strong need to identify the habitat of adult females [6]. In this regard, Darwin Island constitutes a unique opportunity to carry out these studies in order to provide essential information to understand their reproductive cycle, a fact that might be essential for the species' conservation.
